# *Lechebnaia pedagogika*: The Concept and Practice of Therapy in Russian Defectology, c. 1880–1936

**DOI:** 10.1017/mdh.2017.76

**Published:** 2018-01

**Authors:** Andy Byford

**Affiliations:** MLAC, Durham University, Elvet Riverside, New Elvet, Durham DH1 3JT, UK

**Keywords:** Russia, USSR, defectology, therapeutics, children

## Abstract

Therapy is not simply a domain or form of medical practice, but also a metaphor for and a performance of medicine, of its functions and status, of its distinctive mode of action upon the world. This article examines medical treatment or therapy (in Russian *lechenie*), as concept and practice, in what came to be known in Russia as defectology (*defektologiia*) – the discipline and occupation concerned with the study and care of children with developmental pathologies, disabilities and special needs. Defectology formed an impure, occupationally ambiguous, therapeutic field, which emerged between different types of expertise in the niche populated by children considered ‘difficult to cure’, ‘difficult to teach’, and ‘difficult to discipline’. The article follows the multiple genealogy of defectological therapeutics in the medical, pedagogical and juridical domains, across the late tsarist and early Soviet eras. It argues that the distinctiveness of defectological therapeutics emerged from the tensions between its biomedical, sociopedagogical and moral-juridical framings, resulting in ambiguous hybrid forms, in which medical treatment strategically interlaced with education or upbringing, on the one hand, and moral correction, on the other.

## 1 Introduction: Defectology between Diagnostics and Therapeutics

Therapeutics is the domain of medical practice that has historically been particularly difficult to subordinate to modern rationality, to systematise as an area of scientific knowledge, and to secure as a territory under the full control of medical authority.^1^ It is in the realm of therapy that physicians have had to contend with all manner of rival practitioners and alternative, folk or lay healers. Yet therapy is not simply an area or form of medical practice (heterogeneous and contested as it might be); it is also a metaphor for and a performance of medicine as such – of its functions and status, of its distinctive mode of action upon the world. The framework of ‘therapy’ generated in medicine is regularly ‘borrowed’ in order to lend specific meanings and legitimacy to otherwise highly diverse contexts of healing and curing, treatment and relief, restoration and rehabilitation. At the same time, the metaphor and performance of ‘therapy’ have allowed medical professionals themselves to extend their authority and practice to areas beyond those associated strictly with the treatment of illness and disease.

This article examines one such case, namely the place that ‘therapy’ – in Russian *lechenie* – occupied as both concept and practice in what came to be known in Russia as ‘defectology’ (*defektologiia*). Defectology was the discipline and occupation concerned with the study and care of children with developmental pathologies, disabilities and special needs. Its origins are to be found in late tsarist Russia, but it became fully institutionalised under this name only in the early Soviet period.^2^ The pioneers of the field were typically doctors, mostly those specialising in hygiene, psychiatry and neurology, who nevertheless strategically positioned their expertise across medicine’s boundaries with education and psychology.^3^ In the Soviet Union defectology crystallised into the main disciplinary structure for special needs education, recruiting those with training in psychology and pedagogy even while retaining clinical approaches and therapeutic models deriving from medicine.

During the 1920s, defectology was closely associated with the broader, multidisciplinary, field devoted to the study of child biopsychosocial development, which in the early Soviet Union thrived under the name ‘paedology’ (*pedologiia*).^4^ In the mid-1930s however, paedology was condemned by the Communist Party as a ‘pseudo-science’, not least because it was deemed to have unduly over-diagnosed subnormality in the Soviet child population, especially among the working classes and ethnic minorities, mostly by the unwarranted use of mental testing.^5^ Defectology, whose remit at this point became restricted to the clinical establishment and treatment of concrete ‘impairments’, remained in place as an autonomous and more narrowly defined occupational specialism.^6^ It continues to exist under this name in today’s Russian Federation.^7^

The trans-professional character of defectology was explicit in its self-presentation from the earliest days. The term *defektologiia* became dominant only in the 1920s and initially this specialism was referred to as ‘medical pedagogy’ – in Russian, variously, *meditsinskaia, vrachebnaia, patologicheskaia* or *lechebnaia pedagogika*. These different labels were used interchangeably and synonymously, the adjectives ‘meditsinskii’, ‘vrachebnyi’, ‘patologicheskii’ and ‘lechebnyi’ all connoting medical expertise. Individually, however, these qualifiers pointed to different semantic facets of medicine. The first, *meditsinskii*, of Latin provenance, invoked medicine in the most general of ways; the second (from the word *vrach*, etymologically synonymous with *znakhar’* – witchdoctor) emphasised medicine as a special (secret, hidden, magical) form of knowledge and skill. The third and fourth highlighted, respectively, two different, if complementary, aspects of medical expertise. The former (*patologicheskii*), invoking ‘pathology’, flagged medicine’s *diagnostic* dimension, its power to name and identify what was ‘wrong’, ‘anomalous’ or ‘defective’. The latter (*lechebnyi*) stressed medicine’s *therapeutic* dimension, its power to treat and heal.^8^ It is this last aspect of defectology that this article will focus on.^9^ While it is not possible to extricate the therapeutic dimension from the diagnostic one, since forms of treatment usually depend on the conception and identification of a given pathology, this distinction is important. The power to diagnose and the power to treat represent different bases for ‘professionalism’ in terms of the relationship that is established between practitioner and client.^10^

Looking at the period between the 1880s and the 1930s, and using a range of published sources (articles from professional journals, occupational manuals and scientific studies of this era), as well as a number of archival collections, I will examine how defectological therapeutics arose and evolved in the turbulent social transformations that Russia experienced across the late Imperial and early Soviet periods.^11^ In particular, I will follow its multiple genealogy in the medical, pedagogical and juridical domains, situating it in the occupational niche focused on children considered ‘difficult to teach’, ‘difficult to cure’ and ‘difficult to discipline’.

These distinct yet overlapping areas of professional work were small and marginal, disparate and fragmented, avoided rather than coveted – both in the broader social context and within the respective fields of education, medicine and law. The marginality and heterogeneity of this zone allowed it to be (partially and imperfectly) ‘medicalised’, since medicine at the time offered the most developed rational discourse for normatively articulating ‘the wrong’ in human life. What is more, defectological therapeutics was the principal form of expertise that claimed ‘the wrong’ for itself (rather than merely identifying and then expulsing it).^12^ At the same time, however, the nature of the ‘imperfections’ to be ‘treated’ in this context was by no means straightforward to pin down.^13^ Distinctions between very broad categories of ‘imperfection’, such as the blind, the deaf, the invalid, and those with learning difficulties or behavioural issues might have been relatively unambiguous in and of themselves.^14^ However, the full range of ‘imperfections’ was very broad and, crucially, in diagnostic practice, physical anomalies, sensory impairments, psychopathologies and problems of cognitive function were commonly treated as interlinked, co-constitutive and requiring treatment within an overarching ‘medico-pedagogical’ framework.^15^

Consequently, Russo-Soviet defectology offers the case of an impure therapeutic field – one which arose historically between different types of expertise, resulting in ambiguous, hybrid forms of ‘therapy’ where medical treatment interlaced with education or upbringing, on the one hand, and moral correction, on the other. What I will argue in what follows is that the distinctiveness of defectological therapeutics (of its meanings and its effects), can be found in the semantic tensions generated when these different frameworks of intervention – the biomedical, the sociopedagogical and the moral-juridical – are made to metaphorically re-describe each other, whether in defectology’s professional and disciplinary rhetoric or in the very performance of concrete therapeutic acts themselves.^16^

## 2 Therapy on the Boundaries of Medicine and Education

The emergence of the Russian field of defectology was part of broader processes of medicine’s jurisdictional expansion typical of the late nineteenth and early twentieth centuries. Doctors were at this time moving increasingly outside their professional safe-havens – those of physical pathology – to claim many other aspects of human life, individual as well as social. This was especially conducive to the development of social medicine in its many forms, from the hygiene movement to eugenics.^17^ Expansion from the clinical emphasis on pathology to the prophylactic focus on health, and from the traditional, secure domain of the body to the less tangible realm of the soul were the most significant strategic moves in this context. Medical practitioners were here often entering spaces and engaging with phenomena simultaneously claimed by other occupations. One such realm was that of childcare and education, understood as *the* social institution responsible for nurturing future generations.^18^ From the 1860s onwards, with the speeding up of Russia’s modernisation following the Great Reforms of Alexander II, some parts of the medical profession in Russia sought to play a more elaborate role in this domain.^19^

Although the concept and promise of ‘health’ (*zdorov’e*; *ozdorovlenie*) were the watchwords of medical expansion into various social spheres, including education, it was through pathologisation that doctors accessed these other territories most successfully. Medical professionals regularly critiqued the traditional methods of upbringing, teaching and disciplining children that parents, nannies and teachers relied on, arguing that these could become the primary causes of physical and psychological pathology.^20^ At times, however, Russian doctors also flattered teachers and educated parents that they too should be able to form what they referred to as ‘pedagogical diagnoses’ – that is, to at least recognise particular problem conditions encountered in child development.^21^ And what is more, when speaking to parents of such problem children, they sometimes argued that the boundary between therapy (*lechenie*), as the remit of doctors, and upbringing (*vospitanie*), as the remit of parents, was, in fact, a fuzzy one.^22^

While the domains of infant care and early upbringing (populated by parents and nannies) were readily accessible territories of influence, when it came to formal education, doctors needed to negotiate a form of jurisdictional division of labour with the teaching profession. In the context of regular schooling, this was by no means easy. For instance, efforts by Russian pioneers of school hygiene to expand the role of the school doctor and strengthen his influence on day-to-day education was largely resisted by teachers.^23^ Ultimately, the school doctor’s functions in tsarist Russia’s schools remained auxiliary, restricted mostly to routine health checks and elementary anthropometrics.^24^

The more extensive and successful incursion of medical professionals into educational territory occurred in the creation of facilities for the care and education of children who came to be defined as, in one way or another, ‘defective’.^25^ This was, by definition, a marginal zone in tsarist Russia’s rather rigid state education system – a zone populated by children considered ‘difficult to teach’ (*trudnovospituemye*), who were being expulsed from regular schooling, or at best referred to alternative facilities, if and where these were available. Due to more rapid educational expansion in the first decades of the twentieth century – tentative in the late tsarist era and then all-encompassing in the early Soviet Union – this zone not only grew in size, but shifted centre-stage, in some respects even partially fusing with ‘normal’ schooling, especially during the 1920s.^26^ It was in this niche that medical professionals successfully claimed for themselves those sub-areas of education in which teachers were, in fact, quite happy to pass responsibility to another occupation. It was here that defectology as a discipline and occupation arose and boomed.

In late tsarist Russia there were three different types of establishment that served as institutional pillars of this emerging field.^27^ First, there were a few relatively small private medico-pedagogical sanatoria – for example, those of Ivan V. Maliarevskii in St Petersburg and Vsevolod P. Kashchenko in Moscow (to be discussed in what follows).^28^ These were set up and run by figures with medical degrees, but the daily care of and work with children was carried out mostly by staff without medical qualifications. Second, there were charitable shelters run by lay female philanthropists, built largely on a religious foundation (the most developed being E.K. Gracheva’s network).^29^ Funded by private donations, they housed the largest number of children. Several neurologists and psychiatrists (eg. V.M. Bekhterev, A.S. Griboedov, P.I. Kovalevskii and G.I. Rossolimo) found it convenient to base part-time clinics at such shelters.^30^

Finally, there was a growing number of so-called auxiliary classes and schools (*vspomogatel’nye klassy i shkoly*, a term based on the German *Gehülfschulen* and *Hilfsschulen*).^31^ At the planning stages, in the 1890s, these were imagined as extensions of psychiatric clinics and were supposed to be headed by a ‘physician-psychiatrist’ who would be working ‘in close co-operation with a specialist-teacher’.^32^ However, *c*.1910, auxiliary classes and schools arose, in fact, out of the primary school system and were run by local educational administrations. Nonetheless, precisely because of the close link between such schools and regular education, the distinction between ‘normality’ and ‘pathology’ became particularly important here and doctors assumed a critical role in patrolling this boundary.^33^ While auxiliary classes and schools were managed by teaching staff, doctors were essential in the selection of students for them through clinical examinations and tests. In sum, therefore, ‘medicalisation’ seemed most effective on the research, diagnostic and health-monitoring side, but rather less so on the side of treatment and care. More systematic ‘therapeutics’ were being developed primarily in the first type of institution – the school-sanatoria that were actually run by doctors.^34^ These will be examined in greater detail below.

Given the underdevelopment of pedagogy as an academic subject or science (*nauka*) in Russia, doctors were also successful in infiltrating the growing network of new teacher-training institutions that were appearing on the scene in the last couple of decades of tsarism, assuming a prominent role in them as lecturers and consultants.^35^ While doctors often sought to influence pedagogy in an all-encompassing way, their actual specialism was the subject dubbed ‘pedagogical pathology’ (*pedagogicheskaia patologiia*, derived from the German equivalent – *pädagogische Pathologie*).^36^ It was taught mostly by psychiatrists with interest in child psychopathology and it was promoted simultaneously (yet differently) to both doctors and teachers. As part of medical training, that is, as a specialism deemed essential to doctors who were going to work in schools, charitable shelters or facilities for juvenile delinquents, pedagogical pathology was conceptualised analogously to the role of forensic medicine: while the latter was relevant to medicine’s expansion into legal territory, the former concerned its incursion into the realm of education.^37^ This meant that in the medical context emphasis was again placed more on diagnostics than therapeutics, since, in practice, the doctors’ role in schools and shelters was primarily to monitor general health, identify particular pathologies and offer clinical consultations.

However, though taught by doctors, pedagogical pathology became arguably better established as a subject within teacher training.^38^ It was promoted as essential to the expertise of the education profession, since all teaching staff were expected, at some point, to encounter deviant behaviour, physical pathology or psychological defects. Teachers needed to be able to correctly identify these and then act upon their findings, for example, by referring the pupil to the school doctor or by recommending him or her for transfer to a special school. Moreover, the teachers’ pedagogical role was itself expected to include the active development of ‘healthy’ behaviour in their pupils. However, pedagogical pathology was hardly equipping ordinary teachers with formal diagnostic powers (which remained with the doctors), while the idea that teachers needed to be conscious of promoting ‘health’ tended to be framed prophylactically rather than therapeutically: it was more about enforcing hygienic practices than developing models of special education as forms of ‘pedagogical treatment’.

## 3 Juvenile Correction at the Roots of Defectological Therapeutics

Another key origin of Russian defectology, genealogically vital to the conceptions and practices of *lechenie* in this field, was the domain of juvenile criminality and delinquency. This was, likewise, a territory populated with ‘difficult’ children, but the difficulties that this group posed were framed not merely as psychopedagogical, but also as moral-juridical in nature. Some attempts to medicalise juvenile delinquency had begun already within tsarist Russia’s emergent network of correctional facilities for young offenders (*ispravitel’nye zavedeniia*), which included shelters (*priuty*) and colonies (*kolonii*).^39^ Though formally overseen by the ministries of first the Interior and then Justice, tsarist Russia’s juvenile correctional facilities were run philanthropically and thus lacked an established bureaucratic or occupational structure, while its staff remained poorly professionalised. This meant that doctors could, in principle, have had more effective influence here than in schools. However, the medicalisation of juvenile delinquency was, in fact, ambivalent in these institutions as well, especially at first. What is more, it took place mostly in criminological *theory* (namely, in the criminal anthropology of Dmitrii A. Dril’) and tended to be limited to psychiatric classifications of young offenders as belonging to particular psychopathological categories. This happened quite naturally since psychiatric discourse at this time fused medical and moral concerns by including in its diagnostic spectrum forms of ‘depravity’ (*porochnost’*) and ‘deficiencies of character’.^40^

The practical involvement of medical professionals in tsarist facilities for young offenders was in reality minimal. Some larger shelters and colonies had a resident doctor and smaller ones would be visited by one relatively regularly.^41^ However, there was little attempt to medicalise the processes and practices of juvenile correction itself. In fact, in the late nineteenth century, when these facilities were only starting to appear, the medicalisation of correction was explicitly resisted. This was due to the fact that the bulk of inmates at these facilities came from peasant and other labouring strata, and the aim was mostly to reform them into productive and obedient workers.^42^ At best they were to be civilised by disciplinary forms of moral upbringing (*vospitanie*), especially those that involved labour; they were hardly expected to be given therapy. These institutions were still considered forms of detention and places of punishment, which precluded treating inmates as deserving of such privilege as therapeutic attention and special medical or pedagogical care, even when some of them might have been perceived as (criminologically interesting) psychopathological cases. The representation of inmates of juvenile correctional facilities as peasant lads (rather than a horde of the déclassé, which became more common later, with the intensification of urbanisation and industrialisation at the turn of the twentieth century) went hand in hand with their *de-*pathologisation.

An illustration of this, as well as a demonstration of the direct historical link between the field of juvenile criminology and defectology, is evident in the example of the career and work of Dr Ivan Maliarevskii. In the late 1870s Maliarevskii began work both as a doctor (*vrach*) and as an educator (*vospitatel’*) at the St Petersburg agricultural colony for young offenders.^43^ He had originally been a primary school teacher, but went on to complete a medical degree. At the above correctional facility Maliarevskii campaigned vigorously to medicalise the regime of the colony, believing that crime often had as its cause pathological deviations from the correct psychological and physical development. However, his ideas were not accepted by the colony’s board, which argued that the percentage of the feebleminded, psychopaths and invalids at this institution was very small – the majority were healthy children, originally peasants or lower-class townspeople (*meshchane*), who had simply been poorly raised, developed bad habits, and became difficult to control.

This failure led Maliarevskii to leave the colony and to found, in 1882–83, on the outskirts of St Petersburg, Russia’s first special school – a private institution that required parents to pay for the specialist care given to their children.^44^ Maliarevskii defined it as a ‘medico-educational establishment’ (*vrachebnovospitatel’noe uchrezhdenie*) and implemented exactly what he was proposing in his earlier post, only now his target were not young offenders, but a smaller group of children with a range of different problems, from ‘retardation’ to ‘deficiencies of character’ to epilepsy. The establishment’s brochure explained that parents often failed to recognise the ‘deeper’ causes of their children’s problem behaviour – those which were, according to Maliarevskii, rooted in ‘heredity’ (*nasledstvennost’*) and ‘degeneration’ (*vyrozhdenie*), and which therefore required the proper medical assessment, supervision and treatment that his establishment provided. Maliarevskii claimed that he and his staff carefully tracked the development of each child’s body and mind, in order to catch the first signs of ‘pathology’, combating it from its earliest manifestations.

In other respects, the establishment’s objectives were not very different to those of a juvenile correctional facility – namely to turn these children into useful members of society, especially through manual labour, or, ideally, to enable them to return to normal schooling. Maliarevskii’s establishment eventually acquired several houses with gardens which were used for light agricultural work. While some children attended this establishment only for brief periods, others stayed for as long as five years, and a few even remained as adults to work on a nearby farm where their parents bought a plot of land for them. The way Maliarevskii promoted his school at the first Hygiene Exhibition in St Petersburg in 1893 was close to the way juvenile correctional facilities promoted themselves at similar events – by exhibiting the artefacts and other work produced by inmates as artisans.^45^ Therapy itself was based on a schedule of daily routines, especially labour ones, a diet regime and a programme of lessons. Maliarevskii accepted children with very different types of abnormalities and divided his establishment into a ‘medical section’ for those needing permanent supervision, such as epileptics, and an ‘educational section’ for those with lighter ‘retardation’ or behavioural problems. In both cases, though, therapeutic emphasis was on ‘strengthening the organism’, not least by physical work.

As regards the domain of juvenile delinquency itself, it continued to be an important source for what would eventually become defectology. In the early twentieth century, the new criminological discourse presented young offenders as products of the pernicious side-effects of industrial capitalist modernisation – as individuals who, by being taken out of their natural labouring condition, were placed on a path of ‘degeneration’. Criminology at this juncture saw an increasingly active involvement of psychiatrists, who were quite successful in medicalising the discipline – a development that extended into early Soviet discourses surrounding social deviance in juveniles.^46^ Medical pathologisation, driven by criminal anthropology, had to contend, however, with the rival sociological school of criminology rooted in ‘moral statistics’, which was led by M.N. Gernet.^47^ Nonetheless, these two approaches, even while foregrounding distinct causal explanations of crime, were not incompatible. They were, in fact, adopted side by side in early Soviet campaigns to deal with mass delinquency caused by years of wartime and revolutionary upheaval, trauma and displacement.^48^

Indeed, in the aftermath of the First World War, the 1917 revolutions and the Civil War (1917–22), the issue of juvenile criminality was turned into a vast area of state intervention in the context of early Soviet measures to tackle the problem of the millions of homeless waifs, orphans and delinquents, the so-called *besprizorniki* (lit. ‘the unsupervised’).^49^ The Bolsheviks were keen to mobilise all expertise available to them, including, prominently, that of medical professionals already working in this domain. Article 7 of the decree of the Soviet government (Sovnarkom) of the 4th March 1920 stated that ‘The upbringing, training and treatment (*lechenie*) of morally defective juveniles accused of socially dangerous acts is a medico-educational task carried out by the People’s Commissariats of Education and Health in suitable medico-educational establishments (*lechebno-vospitatel’nye uchrezhdeniia*), to which they are to be referred by the Commissions for Juvenile Affairs.’^50^

These developments became crucial to the expansion of defectology in the 1920s, since the campaign to eradicate the phenomenon of *besprizornost’* came to overlap, in practice as well as rhetoric, with the ‘struggle against defectiveness’ (*bor’ba s defektivnost’iu*).^51^ In the first half of the 1920s, the *besprizornik*, framed as ‘moral defective’ (*moral’no defektivnyi*), became defectology’s model subject.^52^ The notion of ‘moral defectiveness’ came to be used very broadly to include a whole range of different kinds and levels of deviance and pathology, leading to a variety of possible ‘treatments’ that the Commissions for Juvenile Affairs could recommend.^53^ At the First All-Russian Conference for the Struggle with Child Defectiveness in 1921, different types of care institutions were proposed for the *besprizorniki*, depending on which category of ‘difficulty’ they belonged to – on how they responded to educational measures, whether they required isolation, how much medical, especially psychiatric expertise, was necessary, and how difficult they were to manage and discipline.^54^ In most cases it was the regime of daily life at the institution that was deemed to have the core therapeutic effect, the idea being especially to rebalance the delinquent child’s ‘will’ (diagnosed as over- or under-developed), that is, to develop self-control, on the one hand, and motivation for work, on the other.^55^

## 4 Hybrid Therapeutics: *Lechenie, Vospitanie, Ispravlenie*

What should be clear from the above is that the field of defectology arose simultaneously out of a number of different, if overlapping, contexts, and that its therapeutic horizon was, as a consequence, fundamentally hybrid. It was made up of three distinct, yet historically interwoven, genealogical strands – the medical, the pedagogical and the juridical.^56^ The concept of *lechenie* (medical treatment) was ambiguously interwoven with that of *vospitanie* (education, upbringing) as well as that of *ispravlenie* (moral correction). In both the discursive representations and the institutional practices of defectological therapeutics, these three concepts flowed easily in or out of one another – both metaphorically, in the sense that each could be used to re-describe the other, and performatively, in the sense that the meanings and effects of particular practices as ‘therapy’ were being produced in and through the therapeutic acts themselves.

The ambiguities created by this hybridity were sometimes beneficial to the field, allowing it to adapt to different conditions and contexts, and to expand accordingly to new ‘supports’ (in the Foucauldian sense of this term – *points d’appui* in French). At other times, hybridity led to internal contradictions and divisions, such as those between doctors and educators, which stalled disciplinary self-realisation around a more coherently defined occupational identity and professional structure. One could say, however, that the distinctiveness of defectological therapy lay precisely in the *tensions* between its biomedical, sociopedagogical and moral-juridical framings.^57^

From the medical perspective, defectological therapy amounted mainly to sanatorium-like regimes of physical and mental hygiene, that is, to ‘soft’, non-intrusive and non-pharmacological, kinds of therapeutics more typical of prophylactic or palliative care. It was akin to therapy used in poorly determined chronic pathologies, where the idea of ‘cure’ was by no means unequivocal. The goals of such therapy tended to be rather vague, amounting to the ‘strengthening of the organism’ or the ‘recovery of the health of one’s personality’ (in Russian – *ozdorovlenie lichnosti*). The term *ozdorovlenie* (lit. ‘achieving health’) was used loosely and metaphorically, rather than as an expression of a clearly specified objective, since what was understood as ‘health’ remained, by definition, open-ended, serving mostly as an expression of a normative value.^58^ Thus, just as from the educational perspective the targets of intervention were those considered ‘difficult to teach’, so from the medical perspective they were, in fact, those ‘difficult to cure’ (at least by the standard repertoire of educational and medical tools respectively). However, the fact that there did not seem to be a definitive ‘cure’ opened up opportunities for therapeutic innovation; and innovation came especially from the cross-fertilisation of concepts and practices between and across the above-described domains of *lechenie, vospitanie* and *ispravlenie*.

Arguably the most significant establishment relevant to the rise of defectology in the tsarist era, which continued and expanded its operations in the early Soviet period, was the School-Sanatorium for Defective Children, founded by Vsevolod P. Kashchenko in Moscow in 1908.^59^ This was the first institution in Russia in which the adjective ‘defective’ was used to describe child developmental pathology in a general way, rather than as a more narrowly defined condition. Previously, the term was used mostly by psychiatrists assessing children in charitable shelters or facilities for young offenders to refer to a specific deficiency, a deficiency imagined as an inherent (‘organic’) lack – the absence of a moral core in the case of ‘moral defectives’ or the deficiency of cognitive processing mechanisms in the ‘mentally defective’. What Kashchenko meant by ‘defective’, though, was broader and vaguer. His school admitted children with a wide variety of ‘imperfections’, but mostly those that went under the labels ‘low-achieving’ (*malouspevaiushchie*), ‘nervous’ (*nervnye*) or ‘difficult’ (*trudnye*).^60^ These ‘defects’ were said to be, by and large, ‘temporary’ and caused not by deep-seated ‘heredity’, but by the harmful influence of the children’s homes and schools.

The aim of Kashchenko’s sanatorium was *ozdorovlenie lichnosti* (‘recovery of the health of one’s personality’) through the ‘re-education’ (*perevospitanie*) of both body and mind.^61^ The classes were small and there was no compulsory school syllabus. Kashchenko divided inmates into ‘families’ – groups that did everything together, each of which had a separate teacher (a practice characteristic of several shelters and colonies for juvenile delinquents). Teachers performed regular tests on the children and used these also as educational activities, thus combining diagnostics with therapeutics.^62^ Emphasis was again placed on manual work, but framed as ‘learning by doing’ (for example, by making models of objects or artefacts such as toys and household items). This was not intended as initiation into an actual artisanal occupation (as would have been the case in facilities for young offenders or at Maliarevskii’s school), but as a form of craft therapy. Other curative/correctional measures included massage, gymnastics, walking, swimming, sunbathing and a compulsory hour-long rest on a deckchair outdoors, even in mid-winter. Children were expected to eat in silence as a way of exercising self-control. Diet was carefully thought through (for example, suppers were without meat and extra doses of milk were given to the weak). The daily routine was meticulously structured, right down to when the children were expected to have cold and when hot showers.^63^

An original medical regimen was used as a disciplinary measure at Kashchenko’s school. If a child did something that was forbidden, he or she would be told that they had done this because they were ‘sick’ and they would be asked to spend some time in bed to recover. This was said to work both medicinally (giving the child’s nervous system a rest) and also pedagogically (in the sense that the child would want to ‘recover’ and would therefore not commit the offence in the future).^64^ Thus, the juxtaposition of the medical, the pedagogical and the correctional was here achieved not through the diagnostic pathologisation of the child and its behaviour as ‘sick’, but in the performance of ‘therapy’ itself.

## 5 The Defectologist as Therapist

The early Soviet era saw a considerable expansion of institutions for ‘defective’ children. The initial network was formed out of the few already existing establishments, which were voluntarily nationalised, with pioneers and former owners, like Kashchenko, remaining in place as their heads. Kashchenko’s school-sanatorium was in 1921 reorganised into the Medico-Pedagogical Station (Mediko-Pedagogicheskaia Stantsiia; MPS) of the People’s Commissariat of Education (Narkompros).^65^ There were, however, many new institutions emerging on the scene, especially in the context of the struggle with *besprizornost’*.^66^ A particularly prominent establishment in Leningrad was the Child Diagnostic Institute (Detskii Obsledovatel’skii Institut; DOBI), run by the psychiatrist Adrian S. Griboedov.^67^ With the expansion of the field at this juncture, there was increased demand for ground-level staff, as well as continuous calls to form a better trained and more specialised occupational group in this particular area of work.^68^

In the early Soviet era, the Commissariats of Education (Narkompros) and Health (Narkomzdrav) were expected to share the burden of administering defectological institutions and preparing staff for these.^69^ As a result, there was a fairly clear division between doctors and educators in this field (the *vrach-defektolog* and the *pedagog-defektolog*).^70^ In principle, they were expected to collaborate over both diagnostic and therapeutic duties. However, in practice, the *vrach-defektolog* was the clinician and prescriber of hygiene regimes, while the *pedagog-defektolog* was the one working with the children on a daily basis, deploying measures associated both with hygiene and forms of special education. However, the ‘therapeutic’ role of the defectologist-teacher was not standardised and there were calls throughout the late 1920s and early 1930s for the remit and nature of specialist responsibilities in this occupational domain to be clarified.^71^

As should be clear from the above, such division of labour between medical and pedagogical staff was characteristic of defectology from the earliest days. What is more, it was not dissimilar to the structuring of medical hierarchy more generally, where one commonly finds doctors assuming positions of oversight as those heading institutions, as professors and researchers defining the academic realm, as clinicians doing the rounds, assessing and classifying patients, and then prescribing interventions and therapies. The practice of therapy itself is, however, often passed on to those not qualified as doctors (nurses, therapists, technicians). Indeed, medical history shows that while doctors jealously retained diagnostic powers, they more easily devolved therapeutic ones to subsidiary occupations (for example, physiotherapy, psychotherapy, occupational therapy).^72^ In the case of defectology, this subsidiary occupational group ended up being staff that could simultaneously be defined as (special) educators (*vospitateli*). It is in and through this delegation that *lechenie* would modulate into *vospitanie* and/or *ispravlenie*. Yet defectological therapeutics as such remained ambiguously stretched between and across these different framings.

Significantly, on the side of developing specialists in defectological pedagogy, the *vospitatel’* acquired a *therapeutic function in and of him/herself*. It was argued (by Kashchenko, for instance) that the ‘educator’s personality’ (*lichnost’ pedagoga*) was an instrument of treatment, requiring the *vospitatel’* to have or develop a particular complex of characteristics.^73^ Therapeutically crucial was his or her behaviour, which was in itself expected to have psychological and hygienic impact (referred to in Russian as *psikhicheski-gigienicheskoe povedenie*).^74^ This included knowing how to be friendly with the child, how to organise collective activity, how to be both patient and persistent, how to restrain one’s anger and frustration, and so forth. In some respects, the *pedagog-defektolog* was an ideal teacher.^75^ On the other hand, the defective child was in this context presented not so much as a developing pupil or even as a patient requiring forms of care and treatment, but as a function of the defectologist’s ‘art’. As Kashchenko tellingly put it – the defective child was an instrument ‘on which one must learn how to play and how to draw a sound even when the instrument is broken and sounds out of tune’.^76^ In order to acquire this ability, the *pedagog-defektolog* was expected to study a course in developmental psychology and to be initiated into the principles of *lechebnaia pedagogika* in both theory and practice.^77^

Important for understanding the way in which the defectologists were being formed at this time is that during the 1920s the field grew alongside and, in fact, increasingly *within* (a) the broader framework of paedology, the general science of child biopsychosocial development, and (b) the pragmatic context of early-Soviet educational reforms associated with unprecedented educational expansion.^78^ In fact, while the defectologists’ specialist remit was indeed the ‘pathological’, this ‘pathology’ was being stretched so widely and thinly in the above contexts that the defectologist ended up being trained as a sub-specialism of both the paedologist and the ‘new pedagogue’. The defectologist’s task was not only to deal with the ‘defective’ or ‘difficult’ children housed in special institutions, but also to contribute to the improvement of the ‘normal’ development and socialisation of the (normative) ‘Soviet mass child’ (*massovyi rebenok* in Russian).^79^

The leaders of defectology, such as Kashchenko, claimed that defectological institutions should, in fact, be credited with pioneering some of the flagship progressive-educational approaches that Soviet educational reformers were deploying in regular schools in the 1920s.^80^ This included the new pedagogy’s emphasis on ‘doing’ (on work and creativity), as well as the replacement of traditional, supposedly overly abstract and arbitrary, school subjects with thematic ‘complexes’, which prompted children to learn through the exploration of real-life situations in their surrounding environment. The first couple of decades of the twentieth century were more generally the era of radical educational experimentation and innovation, during which new institutions and techniques of progressive education were mushrooming in Russia, as elsewhere. After 1917, many of these innovations became associated with Bolshevik revolutionary utopianism and harnessed to the mission of building a society on entirely new grounds. While many educational theories and practices were imported from the West, especially the United States, early Soviet reformers also drew on Russia’s native versions of progressive pedagogy, such as the Tolstoyan ‘free education’ (*svobodnoe vospitanie*) movement, led by K.N. Venttsel’ and S.T. Shatskii, which influenced Lenin’s wife, Nadezhda Krupskaia, a key figure at Narkompros.^81^

One needs to place the ‘therapeutic’, sanatorium-like, institutions, such as Kashchenko’s school, into this early twentieth-century experimental-school mix, since defectological establishments contributed actively to the diversification and innovation of educational practices during this time. The experimental methods used in these different establishments were often seen as drawing on similar principles, allowing Kashchenko to argue that defectology amounted to ‘the treatment of the child on the basis of the latest methods of general pedagogy’.^82^ Nonetheless, school-sanatoria were also distinctive in their identification of special education with ‘curative pedagogy’, a notion rooted in the medical metaphor of therapeutics, rather than in the ‘natural’ rights and freedoms of the child, as would have been the case with schools inspired by the ideas and values of *svobodnoe vospitanie*.^83^

One could say, however, that it was a blend of the parallel, yet distinct, traditions of progressive ‘free education’, on the one hand, and defectological ‘curative pedagogy’, on the other – both of which developed on the margins of the educational field in the late tsarist era – that gave shape to some of the key aspects of the radical school reforms of the Soviet 1920s.^84^ And yet, both the concept of (rights-based) ‘freedom’ and that of (medical) ‘therapy’, which lay at the respective cores of these two traditions, were, inherently, values of the liberal bourgeoisie.^85^ As such, they ultimately proved problematic as foundations for the rapid implementation of mass schooling among large populations of children from peasant and working-class backgrounds in the wake of war and revolution. On the surface, they offered considerable promise: first, because they sought to revolutionise the old pedagogical norms and principles associated with the tsarist education system, which was deemed bureaucratic, repressive and discriminatory; and second, because they seemed to be well aligned with core Marxist values, given that they fostered forms of emancipation (of the entire person), not least through labour. Yet the (bourgeois) privileges of both ‘freedom’ and ‘therapy’ had to lose out to the demands of real (mass, industrialised and often forced) labour to which the Soviet nation was ultimately subordinated. This became clear at the end of the 1920s, with Stalin’s ‘Great Break’ and the introduction of the First Five-Year Plan, which led, among other measures, to a radical U-turn in educational policy.^86^ In that turn, and especially from the mid-1930s, defectological treatment, as such, became confined to a pathologised minority – to a consciously *restricted* number of the congenitally ‘defective’, diagnosed with very specific (essentially organic) ‘impairments’ or ‘disorders’ (*narusheniia*), diverse and not always clearly determined as these might still have been.^87^

## 6 Defectological Therapy between ‘Correction’ and ‘Compensation’

In the late tsarist era, doctors who engaged in defectology mostly saw themselves as fighting broadly and vaguely conceptualised ‘degeneration’ (*vyrozhdenie*), which had as its reverse the equally broad and vague notion of ‘attaining health’ (*ozdorovlenie*).^88^ However, in the course of the 1920s, the Bolsheviks’ efforts to build a new society and to enhance, as well as emancipate, the workforce, required a rather more tangible goal.^89^ It now seemed necessary to go beyond the process of protracted, sanatorial ‘treatment’ (*lechenie*) in order to, as rapidly as possible, reach the state of ‘recovery’ (*izlechenie*). Consequently, in the 1920s defectological therapeutics acquired a new conceptual frame that played down palliative treatment and foregrounded the achievement of functional fitness (for schooling and labour, in particular).^90^

The key concept in defectological therapeutics at this time became ‘correction’ (*korrektsiia*), with the dominant strand of defectological practice being labelled ‘corrective pedagogy’ (*korrektsionnaia pedagogika*), a term still in use today.^91^ The idea of ‘correction’, understood as a process of ‘therapy’ oriented towards a quite specific, positive, outcome, was used before the revolution in Kashchenko’s promotion of his school-sanatorium, which, as mentioned, dealt predominantly with cases of learning difficulties and non-standard behaviours that appeared ‘correctible’ through physical and mental hygiene, combined with innovative educational measures.^92^ After the revolution, the Museum of Paedology and Pedagogy of Exceptional Childhood at Kashchenko’s MPS included an entire room devoted to the ‘correction’ of different ‘psychological processes’.^93^ Moreover, in the context of correcting difficult behaviour, ‘corrective pedagogy’ retained also the sense of ‘correction’ that came from the moral-juridical realm, with *korrektsiia* becoming, in part, a modulation of *ispravlenie*.

The idea of ‘correction’ was applied in a fairly unproblematic way to children diagnosed with organic, bodily and sensory-motor, ‘impairments’, such as the blind, the deaf, the physically disabled, or children with speech impediments.^94^ However, the notion was also extended to the treatment of learning difficulties, mental pathology and antisocial behaviour. The meanings of ‘correction’ were capable, in fact, of considerable expansion. What was expected to be ‘corrected’ became very broad indeed – namely, the entire ‘personality’ (*korrektsiia lichnosti* in Russian).^95^

The idea of ‘correction’ was based on an *orthopaedic* model of therapeutics. Early Soviet defectologists, most notably Aleksei N. Graborov (representative of a new generation of leaders in defectology who were not medically trained but came out of late-tsarist teacher-training courses that included initiation into ‘curative pedagogy’) developed and promoted systems of exercises referred to as ‘psychic orthopaedics’ (*psikhicheskaia ortopediia*).^96^ The term ‘orthopaedic pedagogy’ (*ortopedicheskaia pedagogika*) was also used in this context. *Psikhicheskaia ortopediia* echoes Alfred Binet’s ‘mental orthopaedics’, but it actually prioritised sensory-motor development and was thus arguably closer to Maria Montessori’s sensorial exercises. However, what Graborov’s *psikhicheskaia ortopediia* unequivocally shared with Binet’s ‘mental orthopaedics’, was a reliance on ‘orthopaedics’ as a very particular metaphor of therapeutic intervention.^97^

And yet, in early Soviet defectology, ‘correction’ still seemed to include more or less the same repertoire of sanatorial measures of physical, mental and moral hygiene, special-educational methods, and forms of civilising socialisation (framed as *vospitanie*) that had been developed earlier in the context of *ozdorovlenie* (rather than *korrektsiia*) *lichnosti*. For sure, different early Soviet institutions prioritised different approaches, some more educational, others more medical. The working conditions at defectological institutions varied, of course, and the majority, especially those in the more remote provincial areas, suffered from an extreme sparsity of resources, material as well as human.^98^ However, in the more centrally located and better-funded establishments, such as MPS in Moscow, therapy included, as before, a regimen of work and rest, controlled nutrition, gymnastics and breathing exercises, art and games, as well as prescribed periods of peace and quiet to avoid stress (the idea being to remove the child from the ‘unhygienic’ family environment).^99^ At Leningrad’s DOBI, where the framing of defectological interventions was more medical, prescribed treatments also included the use of quartz lamps, electric impulses, hypnosis, psychotherapy and even psychoanalysis (the latter was said to have been used ‘cautiously’, though, and mostly diagnostically: ‘to clarify the sexual constitution, pre-genital organisation and internal conflicts’ in the child).^100^

At the same time, even ordinary schoolwork was presented as a method of ‘correction’ at these establishments.^101^ This encompassed the fostering of ‘work-related creativity’ (*trudovoe tvorchestvo*) insofar as motivating children by ‘creative labour’ (*tvorcheskii trud*) was deemed key to their rehabilitation.^102^ Similarly, collective games and celebrations, singing in a choir or performing in an orchestra were conceptualised as methods of ‘social correction’ (*sotsial’naia korrektsiia*).^103^ However, despite the breadth and looseness with which ‘correction’ was being understood, crucial to defectological therapeutics remained a continued medical framing of such activities – namely, the systematic use of medical metaphors, such as that of administering a particular ‘dose’ of hygiene or labour activities. Thus, at DOBI, emphasis was placed on the ‘paedological grounding of the dose and form of activities’ prescribed as ‘treatment’.^104^ At MPS, the ‘therapeutic effect’ (*lechebnoe vozdeistvie*) of the ‘organisation of a child’s behaviour’ was said to be based on ‘correction, individualisation, and the [appropriate] dosing (*dozirovka*) of study tasks’.^105^

Heterogeneity at the level of therapeutics was also matched by an increasingly broad understanding of ‘defectiveness’ (*defektivnost’*) itself. By the end of the 1920s, this notion came to be used as an umbrella term for an amorphous web of conditions in which a child’s expected development of physical, sensory and mental functions had been (unevenly) affected by a range of different causes, from heredity and trauma to infections and malnutrition to neglect and abuse.^106^ ‘Defectiveness’ was thus understood ambiguously as *both more and less* than ‘illness’ (*bolezn’*); it was a phenomenon that crossed the boundaries (but also maintained the connections) between the strictly medical and the broadly social.^107^ The consequence of this was that while the ‘defect’ itself (understood merely as an outer manifestation of a deeper, but also vaguer, problem) was no longer viewed as meaningfully ‘correctible’ in and of itself, ‘the defectives’ *were* perceived as eminently ‘corrigible’.^108^

This led some, most notably Lev Vygotsky, to question the idea of ‘correction’ targeted on a specific ‘pathology’ as opposed to the person as a whole – and, what is more, the person as situated in a broader sociocultural context. Vygotsky emphasised the importance of reinforcing the ‘healthy’ bodily, mental, and, not least, social functions, which needed to be therapeutically and pedagogically boosted to ‘compensate’ for the defective, pathological, ‘in-correctable’ ones.^109^ In Vygotsky’s framing, the essence of ‘defectiveness’ lay not in some organic or mental malfunction per se (to be ‘corrected’ by physical, sensory or mental orthopaedics), but in the character of relations that connected ‘the defective’ to his or her social environment.^110^ It was on the re-establishment of these relations that ‘compensation’ (*kompensatsiia*) was expected to focus. As a result, Vygotsky stressed ‘social pedagogy’ (*sotsial’naia pedagogika*) over ‘curative pedagogy’ (*lechebnaia pedagogika*), reframing the notion of ‘recovery’ (of health or fitness) into that of (social) ‘rehabilitation’.^111^ And yet, given that ‘correction’ itself had in practice been understood so broadly and loosely, and was able to expand flexibly well beyond the strictly ‘orthopaedic’, or indeed the ‘medical’ more generally, the seemingly opposed notions of *korrektsiia* and *kompensatsiia* did not, in fact, result in radically different models of therapy itself. They both ended up being incorporated into defectological practice as two sides of the same therapeutic coin.^112^

## 7 Conclusion: The Meanings of Defectological Therapeutics

There is little doubt that the treatment of children with developmental pathologies, disabilities and special needs is a subject for medical history: this was a field that, during the late nineteenth and early twentieth centuries, came to be strongly shaped by the medicalisation of developmental norms and the pathologisation of deviations from them. Nevertheless, this article’s analysis has shown that, despite the vital role that medical professionals played in the emergence of Russo-Soviet defectology between the 1880s and the 1930s, the field crystallised through a complex dynamic of interaction between several different areas of professional expertise spread across distinct territories of occupational jurisdiction – the medical, the educational and the juridical.

By focusing on therapeutics, this article emphasised not how defectology pathologised those it identified as its subjects, but how it framed its modes of action upon them. For the rise of defectology as a field of therapeutics, important was less its subjects’ apparent deviations from a normatively framed path of ‘natural’ development and more the fact that the individuals in question did not conform to established occupational modes and powers of guiding child development towards a given normative goal (that of productive, healthy and civilised adulthood). Indeed, the consequence of analysing defectology in terms of ‘therapeutics’ is that its subjects are no longer to be viewed simply as those who were identified as, in one way or another, ‘defective’, but rather, as those who seemed to fall outside recognised frameworks of pedagogical, medical and juridical work – in other words, those categorised as *difficult to educate, cure or discipline*.

Defectological therapy ended up entailing a *hybrid blend* of occupational goals that came from and belonged precisely to these pre-existing occupational domains. In defectology, the goals of educating, curing and disciplining *combined* to form the ‘special’ character of defectological ‘treatment’. The latter became composed of a wide range of measures which were given, simultaneously and overlappingly, medical, pedagogical and moral meanings. I conceptualised this layering as an effect of metaphoric re-description, arguing that the meanings of defectological therapeutics could not be reduced to either of its components, but remained contingent on their juxtaposition, while maintaining semantic tensions between them. That said, the medical ‘layer’ proved particularly important to the institutional rise of defectology. I attributed this to several factors: the greater authority of doctors relative to other key professionals in the field (especially teachers); the dominance of medicine in claiming for itself all manifestations of human life that in one way or another deviated from the norm; and the rhetorical ability of medical discourse to extend beyond biological life to impose itself on a wide range of different social domains.

This is not to say, however, that medicine was able in and of itself to monopolise the way in which defectological practice would be defined and governed. As I have shown, while medical professionals might have dominated clinical practice, defectological diagnostics, and the teaching of pedagogical pathology, when it came to therapeutics, defectology remained thoroughly hybrid. In fact, the doctors who became the true pioneers and leaders of the discipline in Russia were precisely those who were most ready to adopt eclectic forms of practice and discourse in which the curative fused readily and ambiguously with the pedagogical and the correctional.

The reason for this was that medicine did not, in fact, on its own hold all the therapeutic keys. Defectological subjects – whether diagnosed with sensory or physical impairments, learning difficulties or behavioural issues – were not ‘sick’ in the strict sense of the term. And what is even more important, they were not able to straightforwardly ‘recover’ or be ‘cured’. Just as they fell outside the remit of standard educational or juridical powers, ‘the defective’ were not unequivocally susceptible to the powers of medicine either. That was why, in defectology, therapeutic goals needed to be opened up and defined more loosely as a blend of *ozdorovlenie* (the achievement of health) and *perevospitanie* (re-education). The measures of achieving these included a whole array of medical, pedagogical, psychological and disciplinary devices, from mental exercises to craft and agricultural labour; from diet regimes, gymnastics and enforced rest to games and theatre productions; from rules of conduct, like obligatory silence at dinner table, to innovative progressive-educational methods, like learning by doing.

What medical measures were applied within defectological therapeutics tended to be ‘soft’ (hygienic and palliative) in nature. There where therapy appeared more interventionist and targeted, its medical character became less and less literal. This was the case, for example, with the defectological use of concepts such as ‘orthopaedics’ or ‘dosage’, where medical language came to be used largely to describe non-medical practices. Yet the metaphoric use of medical language in such cases should not be understood as sheer rhetorical pretence. The meanings of defectological therapeutics, as occupational practice that strategically occupied the zone on the margins of, in-between or immediately outside established occupational territories, the zone in-between, depended vitally on these metaphoric re-descriptions. Rendering educational or correctional measures ‘curative’ or ‘therapeutic’ was *the* way of circumscribing the zone of ‘special’ interventions targeting those who could *not* be educated, cured or disciplined using standard tools and frameworks. This applies also to the defectological concept of ‘correction’, the metaphoricity of which enabled its expansive application across a highly diverse range of therapeutic practices.

Defectology did not, of course, arise in isolation from innovations taking place across the human sciences or, indeed, within those very occupations in which this field finds its roots (education, medicine and law). Particularly closely aligned to the history of Russo-Soviet defectology was, on the one hand, the history of child study or paedology, and, on the other, the rise of progressive pedagogy. Neither of these was focused principally on the ‘pathological’ in the child population. However, paedology’s task of charting developmental norms could not do without conceptualising deviations from these norms, hence its consistent intertwining with defectology. As for progressive pedagogy, it emerged precisely through the shifting of the boundaries of what ‘normal’ education meant, mostly in the context of the radical expansion and democratisation of education during this key period. Indeed, defectology’s interest in those ‘difficult to educate’ overlapped with progressive pedagogy’s efforts not just to innovate teaching methods, but also to expand the horizons of educational practice to new target populations who would not have previously had access to regular schooling.

The history of Russo-Soviet defectology is inseparable from closely related developments taking place across Europe, North America and beyond. Many of defectology’s early inspirations came from concepts and practices that originated in Western Europe and the United States, although these tended to be adopted eclectically and were reworked to suit local sociocultural and politico-ideological circumstances. However, crucial to defectology becoming what it was, were, unquestionably, the unprecedented social and political transformations that Russia experienced from 1917. This period became the turning point, during which defectology shifted from the margins to the centre of the occupational zone focused on the study and care of the child population in the early Soviet Union. The traumatic effect that societal collapse and years of violence and displacement had on the children of the former Russian empire between the First World War and the end of post-1917 Civil War, combined with the Bolshevik regime’s turning of the entirety of the Soviet child population into a major target of state concern and intervention, generated considerable demand for defectology’s ‘curative pedagogy’, leading to a significant expansion of its occupational infrastructure during the 1920s.

However, once the initial period of dealing with collapse and trauma was over, in the post-revolutionary context of building a new society and workforce, protracted, sanatorial and experimental, treatment seemed less important than the rapid achievement of functional fitness. Consequently, towards the end of the 1920s, defectological therapeutics forged a conceptual frame that played down palliative treatment and foregrounded the goal of targeted recovery. In this context, defectological therapy came to be conceptualised primarily as ‘corrective pedagogy’. The use of labour-related techniques looked especially promising in this respect, thanks to their apparent compatibility with Marxist ideology. At the same time, however, the meanings of ‘correction’ expanded greatly, especially in its transfer to the mental and behavioural sphere. What was to be ‘corrected’ became extremely broad, encompassing the entire ‘personality’. For sure, the notion of ‘correction’ was criticised as inadequate by figures such as Vygotsky, who proposed instead a ‘social pedagogy’ focused on ‘compensation’ – the building up of what was healthy and functional through the reorganisation of the individual’s interaction with his or her social environment. However, in practice, Soviet defectologists continued to rely on eclectic and hybrid therapeutics that intertwined the curative, the pedagogical and the correctional, irrespective of how precisely this was framed theoretically.

In occupational terms defectology remained stretched across, on the one hand, medical professionals, who acted more as researchers, clinicians and diagnosticians (and as such retained the sense of being doctors first and foremost), and, on the other, special educators, who came from teacher-training courses and who were the principal dispensers of defectological ‘therapy’. The defectological therapist was – through this very role, which entailed a distinctive set of idealised personal characteristics and forms of conduct – expected to become, in his or her own right, an instrument of therapy. From the perspective of medicine, the defectologist resembled one of the subsidiary therapeutic occupations; and yet, this group in effect formed an autonomous occupation which remained closely related to the wider teaching profession.

Despite the fact that the allied field of paedology was denounced as anti-Soviet and purged from 1936, specifically on account of apparently over-diagnosing subnormality in the Soviet child population, defectology survived and became, in fact, more clearly demarcated as the discipline and occupation focused on the pathological in child development, leaving the zone of ‘normal’ education to the now ‘reinstated’ pedagogy. One could argue that it was defectology’s ‘medical’ dimension that saved it at this critical point. From 1936 onwards defectological therapeutics was expected to treat relatively narrowly classified and clinically established organic (usually congenital neurophysiological) ‘impairments’ or ‘disorders’, and was to use methods that were framed as a combination of the ‘correction’ and ‘compensation’ of an established ‘defect’ in concrete individuals.

The subjects of defectology remained those difficult to educate, cure or discipline, but there was considerable caution now not to repeat the mass diagnostics of subnormality in the overall schoolchild population typical of paedology between the late 1920s and early 1930s. This did not mean that the numbers of ‘the defective’ could not at times experience a surge, as happened, predictably, in the aftermath of the Second World War. Either way, the Soviet state’s principal objective was to contain deviations from the standards of Soviet development and socialisation and this was to be achieved by the principles of (a) rehabilitating all individuals into productive society, and (b) maintaining the reduction of the fundamental causes of defectiveness to forms of organic pathology found in a limited number of individuals. Irrespective of the many further developments that Soviet defectology experienced as its institutional infrastructure, theoretical frameworks and therapeutic practices evolved in the post-Stalin era, diagnostic containment and therapeutic rehabilitation remained the declared occupational tasks of defectology for the remainder of the Soviet period.

## Footnotes

1.
Charles E. Rosenberg, ‘The Therapeutic Revolution: Medicine, Meaning and Social Change in Nineteenth-Century America’, *Perspectives in Biology and Medicine*, 20, 4 (1977), 485–504; Guenter B. Riesse, ‘History of Therapeutics’, *Clio Medica*, 22 (1991), 3–11; John Harley Warner, *The Therapeutic Perspective: Medical Practice, Knowledge, and Identity in America, 1820–85* (Princeton, NJ: Princeton University Press, 1997).10.1353/pbm.1977.0040331231

2.
On the history of defectology, especially the treatment of cognitive developmental pathologies, see Khananii S. Zamskii, *Istoriia oligofrenopedagogiki* (Moscow: Prosveshchenie, 1980) and Kh.S. Zamskii, *Umstvenno otstalye deti: Istoriia ikh izucheniia, vospitaniia i obucheniia s drevnikh vremen do serediny XX veka* (Moscow: NPO Obrazovanie, 1995). On the treatment of the deaf in Russia and the USSR, see A.G. Basova and S.F. Egorov, *Istoriia surdopedagogiki* (Moscow: Prosveshchenie, 1984) and Claire Shaw, *Deaf in the USSR: Marginality, Community, and Soviet Identity, 1917–91* (Ithaca, NY: Cornell University Press, 2017). For a more recent textbook on the history of special education in Russia, see Nikolai N. Malofeev, *Spetsial’noe obrazovanie v meniaiushchemsia mire: Rossiia*, 2 vols. (Moscow: Prosveshchenie, 2010; 2013). For an English-language overview of the emergence and rise of Russo-Soviet defectology, see William O. McCagg, ‘The origins of defectology’, in William O. McCagg and Lewis Siegelbaum (eds), *The Disabled in the Soviet Union: Past and Present, Theory and Practice* (Pittsburgh, PA: University of Pittsburgh Press, 1989), 39–62. See also Maria Cristina Galmarini-Kabala, *The Right to be Helped: Deviance, Entitlement, and the Soviet Moral Order* (DeKalb, IL: Northern Illinois University Press, 2016), 79–93, 117–33.

3.
See also Andy Byford, ‘Professional Cross-Dressing: Doctors in Education in Late Imperial Russia (1881–1917)’, *The Russian Review*, 65, 4 (2006), 586–616.

4.
Evgenii M. Balashov, *Pedologiia v Rossii v pervoi tret’i XX veka* (St Petersburg: Nestor-Istoriia, 2012).

5.
E. Thomas Ewing, ‘Restoring Teachers to Their Rights: Soviet Education and the 1936 Denunciation of Pedology’, *History of Education Quarterly*, 41, 4 (2001), 471–93; Nikolai S. Kurek, *Istoriia likvidatsii pedologii i psikhotekhniki* (St Petersburg: Aleteia, 2004); Andy Byford, ‘Zagrobnaia zhizn’ “nauki” pedologii: K voprosu o znachenii “nauchnykh dvizhenii” (i ikh istorii) dlia sovremennoi pedagogiki’, *Prepodavatel’ XXI vek*, 1 (2013), 43–54; Andy Byford, ‘The Mental Test as a Boundary Object in Early-20th-Century Russian Child Science’, *History of the Human Sciences*, 27, 4 (2014), 22–58.

6.
Andy Byford, ‘The Imperfect Child in Early Twentieth-Century Russia’, *History of Education*, 46, 5 (2017), 595–617.

7.
A.I. D’iachkov, *Osnovy obucheniia i vospitaniia anomal’nykh detei* (Moscow: Prosveshchenie, 1965); Maria K. Gal’marini [Maria Galmarini], ‘Moral’no defektivnyi, prestupnik ili psikhicheski bol’noi? Detskie povedencheskie deviatsii i sovetskie discipliniruiushchie praktiki: 1935–57’, in I. Kikulin, M. Maiofis and P. Safronov (eds), *Ostrova utopii: Pedagogicheskoe i sotsial’noe proektirovanie poslevoennoi shkoly (1940–80e): Kollektivnaia monografiia* (Moscow: Novoe literaturnoe obozrenie, 2015), 107–51; E.L. Goncharova and O.I. Kukushkina, ‘Defektologiia’, *Al’manakh instituta korrektsionnoi pedagogiki RAO*, 5 (2002), http://almanah.ikprao.ru/articles/almanah-5/defektologija While the old-fashioned and potentially politically incorrect nature of some of defectology’s language, including its very name, is acknowledged today, much of its terminology remains in use and is far from discredited, even while new terms, imported and adapted from current Western discourse, are gaining ground. See Malofeev, *op. cit.* (note 2).

8.
This phrase is closest to the German term *Heilpädagogik*, meaning therapeutic or curative pedagogy. McCagg, *op. cit.* (note 2), identifies the German nineteenth-century *Heilpädagogik* tradition of special education as lying at the roots of Russian defectology, given the influence of German doctors and educators on their Russian counterparts in the pre-revolutionary era.

9.
I have dealt with the role of diagnostics in the history of defectology elsewhere. See Andy Byford, ‘Poniatiia subnormy i patologii v istorii rossiiskoi nauki o rebenke pervoi treti XX veka’, *Voprosy psikhologii*, 1 (2015), 111–22, and Byford, *op. cit.* (note 6).

10.
On the notion of ‘professionalism’, see Terence J. Johnson, *Professions and Power* (London and Basingstoke: Macmillan, 1972), 51–60 (the significance of the ‘diagnostic relationship’ is elaborated on pp. 57–58). On the critical importance of the relationship between doctor and patient for understanding therapeutics, see Rosenberg, *op. cit.* (note 1).

11.
Archival sources used in this article come principally from the Scientific Archive of the Russian Academy of Education in Moscow (Nauchnyi Arkhiv Rossiiskoi Akademii Obrazovaniia; hereafter NA RAO), especially the collections of V.P. Kashchenko (f. 139) and Kh.S. Zamskii (f. 113), as well as those of the Rukavishnikov juvenile correctional facility (f. 115).

12.
Georges Canguilhem, *The Normal and the Pathological* (New York: Zone Books, 1991).

13.
On the mélange of mutually competing normative regimes and modes of diagnostics that were harnessed in the identification of ‘imperfection’ among Russia’s children during the first three decades of the twentieth century, resulting in the plurality, indeterminacy and vagueness of determining what constituted infringements of the norm, see Byford, *op. cit.* (note 6).

14.
For a comparative discussion of the treatment of the most important categories of ‘imperfection’ in the child population in Russia and the USSR, see D. Karoli [Dorena Caroli], ‘Deti invalidy v dorevoliutsionnoi i sovetskoi Rossii’, in Vitalii G. Bezrogov, Ol’ga E. Kosheleva and Mariia V. Tendriakova (eds), *Maloletnie poddannye bol’shoi imperii* (Moscow: RGGU, 2012), 138–96.

15.
McCagg, *op. cit.* (note 2), 47–9, presents this as typical of the ‘holism’ of German *Heilpädagogik*.

16.
On the concept of ‘tension’ that lies at the heart of the rhetorical functions of metaphor, see Paul Ricœur, *The Rule of Metaphor: The Creation of Meaning in Language* (London and New York: Routledge, 2003), 291–3. On the role of rhetoric in medicine more generally see, for example, Judy Z. Segal, *Health and the Rhetoric of Medicine* (Carbondale, IL: Southern Illinois University Press, 2008). On the deployment of medical metaphors in the development of psychology as a discipline, see JoAnne Brown, *The Definition of a Profession: The Authority of Metaphor in the History of Intelligence Testing, 1890–1930* (Princeton, NJ: Princeton University Press, 1992), 76–95. For an anthropological take on ‘performance’ in medicine and healing, see, for example, Effie Gemie-Iordanou *et al.* (eds), *Medicine, Healing and Performance* (Oxford and Philadelphia: Oxbow Books, 2014).

17.
This was, of course, a worldwide phenomenon. See, for example, Daniel Pick, *Faces of Degeneration: A European Disorder, c.1848–1919* (Cambridge: Cambridge University Press, 1989); Nikolas Rose, *The Psychological Complex: Psychology, Politics and Society in England, 1869–1939* (New York: Routledge & Kegan Paul, 1985); Mathew Thomson, *The Problem of Mental Deficiency: Eugenics, Democracy, and Social Policy in Britain c.1870–1959* (Oxford: Clarendon Press, 1998); Sol Cohen, ‘The Mental Hygiene Movement, the Development of Personality, and the School: The Medicalization of American Education’, *History of Education Quarterly*, 23, 2 (1983), 123–49; Julia Rodriguez, *Civilizing Argentina: Science, Medicine and the Modern State* (Chapel Hill, NC: University of North Carolina Press, 2006); Christian Promitzer, Sevasti Trubeta and Marius Turda (eds), *Health, Hygiene and Eugenics in Southeastern Europe to 1945* (Budapest and New York: Central European University Press, 2011). For examples concerning Russia, see John F. Hutchinson, *Politics and Public Health in Revolutionary Russia, 1890–1918* (Baltimore, MD: Johns Hopkins University Press, 1990); Daniel Beer, *Renovating Russia: The Human Sciences and the Fate of Liberal Modernity*, 1880–1930 (Ithaca, NY: Cornell University Press, 2008); Laura Engelstein, *The Keys to Happiness: Sex and the Search for Modernity in Fin-de-Siècle Russia* (Ithaca, NY: Cornell University Press, 1992); Irina Sirotkina, *Diagnosing Literary Genius: A Cultural History of Psychiatry in Russia, 1880–1930* (Baltimore, MD: Johns Hopkins University Press, 2002); Alexander Etkind, *Eros of the Impossible: The History of Psychoanalysis in Russia* (Boulder, CO: Westview, 1997).

18.
On the role of medicine in establishing norms of childhood development, see André Turmel, *A Historical Sociology of Childhood: Developmental Thinking, Categorization and Graphic Visualization* (Cambridge: Cambridge University Press, 2008), 217–35.

19.
Byford, *op. cit.* (note 3).

20.
A classic example of this discourse would be Petr F. Lesgaft, *Semeinoe vospitanie rebenka i ego znachenie* (St Petersburg: Benke, 1910–12). For other examples, see Andy Byford, ‘Roditel’, uchitel’ i vrach: K istorii ikh vzaimootnoshenii v dele vospitaniia i obrazovaniia v dorevoliutsionnoi Rossii, *Novye rossiiskie gumanitarnye issledovaniia*, 8 (2013), http://www.nrgumis.ru/.

21.
E.L., ‘Pedagogicheskaia diagnostika’, *Vestnik obrazovaniia*, 12 (1909), 369–80.

22.
See Elizaveta S. Drentel’n, ‘Lechit’ ili vospityvat’?’, *Vestnik vospitaniia*, 3 (1900), 161–89 (187). This is a speech by Drentel’n, a female psychiatrist, in which she sought to persuade an audience of mothers that one could treat a ‘nervous child’ by pedagogical means.

23.
Byford, *op. cit.* (note 3), 597–607. At the root of the noun *vospitanie* is the verb *pitat’* (to feed or nourish). The term therefore implies ‘nurture’ in a broad sense that includes socialisation, moral instruction, and the inculcation of ‘culture’.

24.
School doctors did, however, play a more important role in extreme cases of ‘pathological’ behaviour, such as suicides of schoolchildren. These were considered to have reached ‘epidemic’ proportions in the wake of the 1905 Revolution. See Aleksandr B. Liarskii, *Samoubiistva uchashchikhsia kak fenomen sotsializatsii v Rossii na rubezhe XIX–XX vekov* (St Petersburg: MIEP, 2010); Susan K. Morrissey, *Suicide and the Body Politic in Imperial Russia* (Cambridge: Cambridge University Press, 2006), 312–45; Andy Byford, ‘Trauma and Pathology: Normative Crises and the Child Population in Late Tsarist Russia and the Early Soviet Union, 1904–24’, *Journal of the History of Childhood and Youth*, 9, 3 (2016), 450–69 (451–4).

25.
Byford, *op. cit.* (note 3), 607–14.

26.
Byford, ‘Poniatiia subnormy i patologii’, *op. cit.* (note 9), 120.

27.
For more detail, see Zamskii, *Istoriia oligofrenopedagogiki*, *op. cit.* (note 2).

28.
Another example would be the Medico-Pedagogical Institute for Underdeveloped, Retarded and Nervous Children, set up in 1904 in Kiev by Olga and Elena Sikorskie, the daughters of Ivan A. Sikorskii, a well-known psychiatrist, who had a strong interest in child development and was one of the pioneers of child study in Russia. *Ibid.*, 285–6.

29.
Zamskii, *Istoriia oligofrenopedagogiki, op. cit.* (note 2), 274–85.

30.
Adrian S. Griboedov, *Nenormal’nye deti v sem’e i shkole: Ocherki po detskoi psikhopatologii i patologicheskoi pedagogike* (Moscow, 1914). Pavel I. Kovalevskii, *Otstalye deti (idioty, otstalye i prestupnye detei), ikh lechenie i vospitanie* (St Petersburg: Vestnik dushevnykh boleznei, 1906).

31.
‘Po voprosu ob organizatsii shkoly dlia umstvenno otstalykh detei’, *Pedagogicheskii listok*, 2 (1907), 93–8.

32.

*2-i s”ezd russkikh deiatelei po tekhnicheskomu i professional’nomu obrazovaniiu. 1896–99: Sektsiia XII, otdel IV* (Moscow, 1898), 33 (quoted in Zamskii, *Istoriia oligofrenopedagogiki*, *op. cit.* (note 2), 259).

33.
For more detail, see Byford, *op. cit.* (note 3), 611–3.

34.
This is not to say, however, that some of the other institutions, especially Gracheva’s shelters, did not contribute to pioneering new forms of special education, most notably the education of those who were both deaf and blind. See T.A. Basilova, ‘Kak nachinalos’ obuchenie slepoglukhikh detei v Rossii’, *Defektologiia*, 2 (1999), 61–3.

35.
Andy Byford, ‘Turning Pedagogy into a Science: Teachers and Psychologists in Late Imperial Russia (1897–1917)’, *Osiris*, 23 (2008), 50–81.10.1086/59186918831318

36.
Ludwig Strümpel, *Die pädagogische Pathologie oder die Lehre von den Fehlern der Kinder* (Leipzig: Boehmes Nachfolger, 1890). See E. Lozinskii, ‘Pedagogicheskaia patologiia’, *Vestnik vospitaniia*, 8 (1910), 106–33, and Adrian V. Vladimirskii, L. Orshanskii and G. Fal’bork (eds), *Voprosy pedagogicheskoi patologii v sem’e i shkole*, 2 vols. (St Petersburg: Shkola i zhizn’, 1912). See also Gracheva’s diary in Zamskii, *Umstvenno otstalye deti*, *op. cit.* (note 2), 355–86.

37.
A.S. Griboedov and N.P. Kazachenko-Trirodov (eds), *Zapiski kratkosrochnykh pedagogicheskikh kursov po podgotovke personala v uchrezhdeniiakh dlia defektivnykh detei* (Petrograd: Kommissariat Sotsial’nogo Obespecheniia, 1918). See also NA RAO f. 139 op. 1 d. 126 ll. 1–2, which describes V.P. Kashchenko’s course in *lechebnaia pedagogika* from 1921.

38.
At the St Petersburg Pedagogical Academy the subject was taught by A.S. Griboedov, at V.M. Bekheterev’s Psycho-Neurological Institute by A.V. Vladimirskii, and at the Moscow Pedagogical Courses by G.I. Rossolimo. See V.P. Kashchenko (ed.), *Defektivnye deti i shkola* (Moscow: Tikhomirov, 1912), 262–3. Major influences in the practice of running specialist facilities for the mentally defective in late tsarist Russia were the Francophone pioneers of this field, namely the Frenchman, Édouard Séguin and the Belgian, Jean Demoor. These authorities were translated in the early 1900s. See Edvar Segen, *Vospitanie, gigiena i nravstvenoe lechenie umstvenno-nenormal’nykh detei*, trans. M.P. Lebedeva, ed. V.A. En’ko (St Petersburg: Likhtenshtadt, 1903) and Zhan Demor, *Nenormal’nye deti i ikh vospitanie doma i v shkole*, trans. Raisa B. Pevzner, ed. Grigorii I. Rossolimo (Moscow: Sytin, 1909).

39.
Catriona Kelly, *Children’s World: Growing up in Russia, 1890–1991* (New Haven, CT, and London: Yale University Press, 2007), 180–92. L.I. Beliaeva, *Stanovlenie i razvitie ispravitel’nykh zavedenii dlia nesovershennoletnikh pravonarushitelei v Rossii (seredina XIX–nachalo XX vv.)* (Moscow: Akademiia MVD Rossii, 1995).

40.
Dmitrii A. Dril’, *Maloletnie prestupniki*, 2 vols. (Moscow: Mamontov, 1884; 1888). D.A. Dril’, ‘Nashi ispravitel’no-vospitatel’nye zavedeniia i voprosy ispravitel’nogo vospitaniia’, *Zhurnal ministerstva iustitsii*, 8 (1898), 173–93; 9 (1898), 88–120; 10 (1898), 101–49; D.A. Dril’, ‘Voprosy ispravitel’nogo vospitaniia’, *Tiuremnyi vestnik*, 3 (1903), 244–59; 4 (1903), 346–57; D.A. Dril’, *O merakh bor’by s prestupnost’iu nesovershennoletnikh* (St Petersburg: Senatskaia tipografiia, 1908). For the broader context, see Pavel I. Bel’skii, ‘Prestupnost’ i deti’, *Psikhologiia i deti*, 1 (1917), 41–51; 2 (1917), 48–56; D.A. Dril’, *Prestupnost’ i prestupniki: Uchenie o prestupnosti i merakh bor’by s neiu* (Moscow: INFRA-M, 2006). S.S. Ostroumov, *Prestupnost’ i ee prichiny v dorevoliutsionnoi Rossii* (Moscow: Izd. MU (Moscow University Press), 1960).

41.
The best example is the Rukavishnikov correctional facility in Moscow. See especially NA RAO f. 115 op. 1 d. 5 ll. 18-21ob. This is a booklet titled *Instruktsii Rukavishnikovskogo priiuta* (1888–89), which includes a brief section on the role of the resident doctor as the one responsible for the facility’s health and hygiene procedures (*sanitarno-gigienicheskaia chast’*). For more on this institution, see M.M. Khin, ‘Moskovskii gorodskoi Rukavishnikovskii ispravitel’nyi priiut dlia maloletnikh prestupnikov (1864–86 gg.)’, *Iuridicheskii vestnik*, 8 (1886), 581–605.

42.
I. Tarasov, ‘K voprosu ob ispravitel’nykh zavedeniiakh dlia maloletnikh prestupnikov’, *Iuridicheskii vestnik*, 12 (1881), 567–76.

43.
On this, see D.T., ‘Ispravlenie maloletnikh prestupnikov v Rossii’, *Iuridicheskii vestnik*, 2 (1885), 325–62, 331–2.

44.
See Zamskii, *Istoriia oligofrenopedagogiki*, *op. cit.* (note 2), 249–52; See also Valentin V. Gorinevskii, ‘Vrachebno-vospitatel’noe zavedenie Dr-a I. V. Maliarevskogo (Iz ekskursii po Pervoi Vserossiiskoi gigienicheskoi vystavke)’, *Vestnik obrazovaniia*, 10 (1893), 328–36.

45.

*Ibid*.

46.
For the late tsarist period, apart from the already quoted works by Dmitrii Dril’, see especially the contributions of Pavel I. Kovalevskii, *op. cit.* (note 30), and *Vyrozhdenie i vozrozhdenie: Prestupniki i bor’ba s prestupnost’iu (sotsialno-psikhologicheskie eskizy)* (St Petersburg: Akinfiev & Lopatov, 1903). For the early Soviet period, see *Deti besprizornye – psikhopaty* (Moscow: Zhizn’ i znanie, 1924); A.S. Griboedov, *Mediko-pedagogicheskaia ekspertiza i komissii po delam o nesovershennoletnikh* (Moscow and Petrograd: Gos. izd., 1924); T.E. Segalov, *Psikhopatologiia imushchestvennykh prestuplenii, sovershaemykh nesovershennoletnimi*, issue 1 (Moscow: Pravo i zhizn’, 1924).

47.
M.N. Gernet, *Sotsial’nye faktory prestupnosti* (Moscow: Un. tip. (Moscow University Press), 1905). M.N. Gernet, *Deti-prestupniki* (Moscow: Znamenskii, 1912). M.N. Gernet, *Moral’naia statistika* (Moscow: Izd. Tsentral’nogo statisticheskogo upravleniia, 1922). M.N. Gernet, *Izbrannye proizvedeniia* (Moscow: Iuridicheskaia literatura, 1974).

48.
Galmarini-Kabala, *op.cit.* (note 2), 82–93; Byford, *op. cit.* (note 24), 456–60.

49.
See Alan M. Ball, *And Now my Soul is Hardened: Abandoned Children in Soviet Russia, 1918–30* (Berkeley, CA: University of California Press, 1994) and Dorena Caroli, *L’Enfance abandonné et délinquante dans la Russie soviétique, 1917–37* (Paris: L’Harmattan, 2004).

50.
Cited in Iurii Iu. Bekhterev, ‘Samoupravlenie kak odna iz form ispravitel’no-trudovogo vozdeistviia na nesovershennoletnikh pravonarushitelei, lishennykh svobody’, *Sovetskoe pravo*, 2 (1926), 121–6 (121–2); translation mine.

51.
Zamskii, *Umstvenno otstalye deti*, *op. cit.* (note 2), 283–301.

52.
See Bekhterev, *op. cit.* (note 50). See also Marina Goloviznina, ‘Politika sotsial’nogo kontrolia prestupnosti nesovershennoletnikh v SSSR (1917-konets 1980-kh gg.)’, *Zhurnal issledovanii sotsial’noi politiki*, 3, 2 (2005), 223–40.

53.
Dorena Caroli, ‘Socialisme et protection sociale: Une tautologie? L’enfance abandonnée en URSS (1917–31)’, *Annales. Histoire, Sciences Sociales*, 54, 6 (1999), 1291–1316: 1296–8.10.3406/ahess.1999.27981621174913

54.
See especially A.S. Griboedov and S.M. Birger (eds), *Spravochnik po voprosam sotsial’no-pravovoi okhrany nesovershenoletnikh i detskoi defektivnosti* (Moscow and Petrograd: Gos. izd., 1924) and V.P. Kashchenko (ed.), *Problemy izucheniia i vospitaniia rebenka* (Moscow: Moskovskoe aktsionerskoe izdatel’skoe obshchestvo, 1926). See also NA RAO, f. 139, op. 1, d. 238, ll. 1–6, which contains minutes from the founding meeting of the Society for the Study and Struggle against Child Defectiveness and *Bezprizornost’* (Obshchestvo izucheniia i bor’by s detskoi defektivnost’iu i besprizornost’iu) of 29 August 1923, chaired by Kashchenko.

55.
Zamskii, *Umstvenno otstalye deti*, *op. cit.* (note 2), 295.

56.
Inter-professional and interdisciplinary interactions were highly relevant in other arenas of occupational work and social action as well. For some examples of areas in which medicine closely interacted with other professional domains in the late tsarist and early Soviet eras, see Frances Bernstein, *The Dictatorship of Sex: Gender, Health, and Enlightenment in Revolutionary Russia, 1918–31* (DeKalb, IL: Northern Illinois University Press, 2007); Sharon A. Kowalsky, *Deviant Women: Female Crime and Criminology in Revolutionary Russia, 1880–1930* (DeKalb, IL: Northern Illinois University Press, 2009); Dan Healey, *Bolshevik Sexual Forensics: Diagnosing Disorder in the Clinic and the Courtroom, 1917–39* (DeKalb, IL: Northern Illinois University Press, 2009); Kenneth Pinnow, *Lost to the Collective: Suicide and the Promise of Soviet Socialism, 1921–29* (Ithaca, NY: Cornell University Press, 2010); Elisa M. Becker, *Medicine, Law and the State in Imperial Russia* (Budapest: Central European University Press, 2011). For a more general sociological account of what is at stake in inter-professional and interdisciplinary collaborations, see Andrew Abbott, *The System of Professions: An Essay on the Division of Expert Labor* (Chicago: University of Chicago Press, 1988), and Michael E. Gorman (ed.), *Trading Zones and Interactional Expertise: Creating New Kinds of Collaboration* (Cambridge, MA: MIT Press, 2010).

57.
The underlying tension here is between the ‘sameness’ and ‘difference’ of *lechenie*, *vospitanie*and *ispravlenie* respectively – a tension which is the property of metaphor itself. See Ricœur, *The Rule of Metaphor*, 232.

58.
The term *ozdorovlenie*was typical of the rhetoric of medicalisation that targeted society in the broadest of ways. Daniel Beer has highlighted its use in criminological and psychiatric discourse on degeneration. See Beer, *op. cit.* (note 17). The same word was used in certain eugenically-minded proposals for school reforms, which called for ‘making the school healthy’ (*ozdorovlenie shkoly*). See V.I., ‘Gimnasticheskie zaly, kak laboratorii legochnykh boleznei’, *Vestnik vospitaniia*, 1 (1894), 247–50. Another way it could be used, was, for example, to argue that a ‘healthy environment’ (*zdorovaia sreda*) could in and of itself re-educate a child. This was made synonymous with the statement that pleasant surroundings and the education of the spirit (*vospitanie dukha*) acted therapeutically on the soul of a ‘defective’ child. See V.P. Kashchenko and S.N. Kriukov, *Vospitanie i obuchenie trudnykh detei: Iz opyta sanatoria-shkoly doktora V.P. Kashchenko* (Moscow: Drukar’, 1913).

59.
V.P. Kashchenko, *Defektivnye deti shkol’nogo vozrasta i vseobshchee obuchenie* (Moscow, 1910). V.P. Kashchenko (ed.), *Defektivnye deti i shkola* (Moscow: Tikhomirov, 1912). I.I. Voskoboinkov, ‘Iz zhizni sanatoria shkoly’, *Vestnik vospitaniia*, 3 (1914), 98–121; V.P. Kashchenko, *Nervnost’ i defektivnost’ v doshkol’nom i shkol’nom vozrastakh: Okhrana dushevnogo zdorov’ia detei* (Moscow: Tseput’kul’t, 1919). Kashchenko, *Problemy izucheniia, op. cit.* (note 54). NA RAO f. 113 op. 1 d. 152 ll. 1–35ob.

60.
Kashchenko and Kriukov, *op. cit.* (note 58).

61.
NA RAO f. 139 op. 1 d. 20, ll. 1–7. NA RAO f. 139 op. 1 d. 6, ll. 23–6.

62.

*Kollektsii spetsial’nykh posobii dlia formal’nogo razvtiia i issledovaniia umstvenno-defektivnykh shkol’nikov i dlia zaniatii v detskikh sadakh* (Moscow, 1912).

63.
Kashchenko and Kriukov, *op. cit.* (note 58).

64.
Zamskii, *Istoriia oligofrenopedagogiki*, *op. cit.* (note 2), 293.

65.

*Ibid.*, 291–2; See also Galmarini-Kabala, *op. cit.* (note 2), 124–9.

66.
Many of them emerged in Petrograd/Leningrad under the auspices of V.M. Bekhterev’s Psycho-Neurological Academy. See A.S. Griboedov (ed.), *Novoe v defektologii* (Moscow: Gos. Psikho-Nevrologicheskaia Akademiia, 1928), and V.M. Bekhterev, ‘Otchet o deiatel’nosti Vospitatel’no-Klinicheskogo Instituta dlia nervnykh detei imeni Akademika V.M. Bekhtereva Psikho-Nevrologicheskoi Akademii’, *Voprosy izucheniia i vospitaniia lichnosti*, 2–3 (1926), 231–4.

67.
E.P. Punina-Griboedova, ‘Desiat’ let defektologicheskoi i pedologicheskoi raboty’, in Griboedov, *ibid.*, 1–17.

68.
On the training of defectologists in the early Soviet era, see A.I. Zhivina, ‘Osnovnye etapy razvitiia sistemy podgotovki uchitelei defektologov v SSSR’, *Defektologiia*, 2 (1974), 68–74, and V. Lapshin and A. Zhivina, ‘60 let vysshego defektologicheskogo obrazovaniia v SSSR i rol’ defektologicheskogo fakul’teta MGPI im. V. I. Lenina v podgotovke defektologov s vysshim obrazovaniem’, *Defektologiia*, 6 (1981), 78–81. On the continuity between pre-revolutionary and Soviet approaches to training in this area, see Griboedov and Kazachenko-Trirodov, *op. cit.* (note 37).

69.
For a heuristic overview of the division of responsibilities between the different Commissariats, see Galmarini-Kabala, *op. cit.* (note 2), 84–7.

70.
On early divisions of labour between doctor and educator in the context of defectology, see Kashchenko, *Nervnost’ i defektivnost’*, *op. cit.* (note 59), 6–15. Kashchenko expressed these two roles more ambiguously as *vrach-pedagog* vs. *pedagog-vrach*.

71.
A.S. Griboedov, A.K. Borsuk and V.V. Belousov (eds), *Voprosy vospitaniia normal’nogo i defektivnogo rebenka* (Moscow and Petrograd: Gos. izd., 1924).

72.
Johnson, *op. cit.* (note 10), 57–8.

73.
V.P. Kashchenko and G.V. Murashev, *Pedologiia v pedagogicheskoi praktike: Pedagogicheskaia klinika* (Moscow: Mediko-Pedagogicheskaia stantsiia, 1926).

74.

*Ibid.*, 14.

75.
The role, personality and conduct of the teacher was also viewed as a vital component of the pedagogical model fostered in Anton Makarenko’s influential method for re-educating juvenile delinquents, exemplified in Nikolai Ekk’s 1931 film *Road to Life* (*Putevka v zhizn’*). See A.S. Makarenko, *Pedagogicheskaia poema* (Moscow: Molodaia gvardiia, 1985). See also: *Vospitanie grazhdanina v pedagogike A.S. Makarenko* (Moscow: Akademicheskii Proekt Al’ma Mater, 2006); E.N. Medynskii, *A.S. Makarenko: Zizn’, deiatel’nost’, pedagogicheskaia sistema* (Moscow: Molodaia Gvardiia, 1944); A.V. Zhilina, N.D. Skvortsova and L.Iu. Gordin (eds), *A.S. Makarenko: K 100-letiiu so dnia rozhdeniia: Ukazatel’ trudov i literatury o zhizni i deiatel’nosti* (Moscow: Pedagogika, 1988).

76.
Kashchenko, *Nervnost’ i defektivnost’*, *op. cit.* (note 59), 9.

77.
Needless to say, this was an ideal rather than a reality, as testified by many poorly trained *vospitateli*in the various children’s homes that had to be established at breakneck speed across a vast territory over the course of the 1920s. See Ball, *op. cit.* (note 49).

78.
On the relationship between paedology and defectology in this era, see Kashchenko, *Problemy izucheniia, op. cit.* (note 54). See also Byford, ‘Poniatiia subnormy i patologii’, *op. cit.* (note 9), 120.

79.
Kashchenko, *Problemy izucheniia, op. cit.* (note 54),153, 162, 172.

80.

*Ibid.*, 168; See also NA RAO f. 113 op. 1 d. 141 l. 11. On the broader context of educational reforms of this era, see E.Iu. Bykova, ‘Reformirovanie sistemy shkol’nogo obrazovaniia v SSSR v 1917–30 gg.: Organizatsionye i ideologicheskie aspekty’, *Vestnik Tomskogo gosudarstvennogo universiteta*, 1 (2011), 179–89.

81.
Konstantin N. Venttsel’, *Novye puti vospitaniia i obrazovaniia detei* (Moscow: Zemlia i fabrika, 1923). See also, Stanislav T. Shatskii, *Izbrannye pedagogicheskie sochineniia* (Moscow: Gos. uch.-ped. izd. Min. Pros. RSFSR, 1958); A.A. Romanov, *Opytno-eksperimental’naia pedagogika pervoi treti XX veka* (Moscow: Shkola, 1997), 139-261; William Partlett, ‘Bourgeois Ideas in Communist Construction: The Development of Stanislav Shatskii’s Teacher Training Methods’, *History of Education*, 2006, *35* (4–5), 453–74.

82.
Kashchenko, *Problemy izucheniia, op. cit.* (note 54), 165.

83.
‘Curative pedagogy’ (*Heilpädagogik*) or ‘curative education’ was also the term that Rudolf Steiner, an influence on Venttsel’, used in his anthroposophical framing of special education. See Rudolf Steiner, *Curative Education: Twelve Lectures for Doctors and Curative Teachers in Donarch from 25th June to 7th July 1924* (London: Steiner, 1972).

84.
Needless to say, early Soviet educational reforms and pedagogical ideas cannot be reduced to these strands. For more detail on this period of educational reformism in the USSR, in terms of policy, practice and pedagogical ideas, see Larry E. Holmes, *The Kremlin and the School House: Reforming Education in Soviet Russia, 1917–31* (Bloomington, IN: Indiana University Press, 1991).

85.
These values are, of course, closely tied to the rise of professions more generally, for which the growth of the middle class, as clients, was crucial. See Johnson, *op. cit.* (note 10).

86.
Larry E. Holmes, *Stalin’s School: Moscow’s Model School No. 25, 1931–37* (Pittsburgh, PA: University of Pittsburgh Press, 1999). Gail Warshovsky Lapidus, ‘Educational strategies and cultural revolution: the politics of Soviet development’, in Sheila Fitzpatrick (ed.), *Cultural Revolution in Russia, 1928–31* (Bloomington, IN: Indiana University Press, 1978), 78–104.

87.
Byford, ‘Poniatiia subnormy i patologii’, *op. cit.* (note 9), 115–8. Byford, *op. cit.* (note 6), 615–7; Zamskii, *Umstvenno otstalye deti*, *op. cit.* (note 2), 332–5.

88.
Kashchenko, *Nervnost’ i defektivnost’, op. cit.* (note 59), 4. For broader context, see Beer, *op. cit.* (note 17).

89.
For broader context, see, for example, Tricia Starks, *The Body Soviet: Propaganda, Hygiene, and the Revolutionary State* (Madison, WI: University of Wisconsin Press, 2008); David L. Hoffmann, *Cultivating the Masses: Modern State Practices and Soviet Socialism, 1914–39* (Ithaca, NY: Cornell University Press, 2011); Raymond A. Bauer, *The New Man in Soviet Psychology* (Cambridge, MA: Harvard University Press, 1952).

90.
Zamskii, *Umstvenno otstalye deti*, *op. cit.* (note 2), 301.

91.
Kashchenko used the variant *korrektivnaia*, rather than *korrektsionnaia*, *pedagogika* He also saw this term as effectively synonymous with *lechebnaia pedagogika* (NA RAO f. 139 op. 1 d. 234 ll. 4–5). For an example of a contemporary textbook in ‘corrective pedagogy’, see N.V. Mal’tseva, *Korrektsionnaia pedagogika s osnovami spetsial’noi psikhologii: Uchebno-metodicheskoe posobie* (Omsk: OOIPKRO, 2009).

92.
NA RAO f. 139 op. 1 d. 20 l. 7. See also V.P. Kashchenko, *Pedagogicheskaia korrektsiia: Ispravlenie nedostatkov kharaktera u detei i podrostkov* (Moscow: Academia, 1999).

93.
NA RAO f. 113 op. 1 d. 141 l. 9.

94.
Kashchenko and Murashev, *op. cit.* (note 73), 15–6.

95.
Kashchenko, *op. cit.* (note 54), 165. NA RAO f. 113 op. 1 d. 152 ll. 7–9.

96.
Zamskii, *Umstvenno otstalye deti*, *op. cit.* (note 2), 299–300. Graborov trained at V.M. Bekhterev’s Psycho-Neurological Institute before the revolution and from the early 1920s headed the Petrograd Pedagogical Institute for the Social Education of the Normal and Defective Child – one of a number of defectological units within the Psycho-Neurological Academy network. He was a major promoter and defender of special schools for the defective. See N.P. Dolgoborodova, ‘Aleksei Nikolaevich Graborov – sovetskii uchenyi-oligofreno-pedagog’, *Defektologiia*, 5 (1972), 82–5.

97.
On the therapeutic metaphor in Binet, see Brown, *The Definition of a Profession*, 82. Binet’s ‘mental orthopaedics’ closely matched his psychometrics. The exercises he recommended were expected to develop (therapeutically) the same functions that the tests measured (diagnostically). ‘Mental orthopaedics’ was vital to Binet’s insistence that the level of mental functioning that his tests measured was not fixed but could be improved by such ‘orthopaedics’. See Stephen J. Gould, *The Mismeasure of Man* (New York and London: Norton, 1996), 176–88.

98.
Zamskii, *Umstvenno otstalye deti*, *op. cit.* (note 2), 298.

99.
Kashchenko and Murashev, *op. cit.* (note 73), 11–23.

100.
Punina-Griboedova, *op. cit* (note 67), 7. See also A.S. Griboedov, ‘Trudnovospituemye deti i piskhoanaliz’, *Voprosy izucheniia i vospitaniia lichnosti*, 1926, no. 1, 57–68.

101.
Kashchenko and Murashev, *op. cit.* (note 73), 20–21.

102.
NA RAO f. 113 op. 1 d. 152 l. 8. NA RAO f. 113 op. 1 d. 141 ll. 9–10. See also V.P. Kashchenko (ed.), *Putem tvorchestva: Stranichka zhizni i opyta odnoi eksperimental’noi shkoly* (Moscow: TsUSTRAN, 1922).

103.
Kashchenko and Murashev, *op. cit.* (note 73), 16–20. NA RAO f. 113 op. 1 d. 141 l. 11. ‘Social correction’ is here contrasted with the ‘natural correction’ (*estestvennaia korrektsiia*) of sensory analysers and motor reactions.

104.
Punina-Griboedova, *op. cit* (note 67), 6.

105.
NA RAO f. 113 op. 1 d. 152 l. 7.

106.
Zamskii, *Umstvenno otstalye deti*, *op. cit.* (note 2), 295. See esp. A.N. Graborov, *Vspomogatel’naia shkola: Shkola dlia umstvenno-otstalykh detei* (Moscow and Petrograd: Gos. izd. tip. im. N. Bukharina, 1923).

107.
Griboedov, *op. cit.* (note 66), v.

108.
Contrast with Michel Foucault’s ‘the incorrigibles’ as a ‘support’ of modern practices of correction, described in his essay ‘The abnormals’, in Michel Foucault, *Ethics: Subjectivity and Truth* (London: Penguin, 2000), 51–57 (52–53).

109.
See Lev S. Vygotskii, *Sobranie sochinenii*, Vol. 5 (Moscow: Pedagogika, 1983), which is devoted in its entirety to Vygotsky’s contributions to defectology. For background on Vygotsky and defectology, see René van der Veer and Jaan Valsiner, *Understanding Vygotsky: A Quest for Synthesis* (Oxford and Cambridge, MA: Blackwell, 1991), 60–77, and Peter Smagorinsky, ‘Vygotsky, “Defectology”, and the Inclusion of People of Difference in the Broader Cultural Stream’, *Journal of Language & Literacy Education*, 8, 1 (2012), 1–25.

110.
Zamskii, *Umstvenno otstalye deti*, *op. cit.* (note 2), 304–7, 311–14.

111.

*Ibid.*, 305, 315. See especially L.S. Vygotskii (ed.), *Voprosy vospitaniia slepykh, glukhonemykh i umstvenno-otstalykh detei* (Moscow, 1924), 16.

112.
Zamskii, *Umstvenno otstalye deti*, *op. cit.* (note 2), 311.

